# The impact of chromium ion stress on plant growth, developmental physiology, and molecular regulation

**DOI:** 10.3389/fpls.2022.994785

**Published:** 2022-10-28

**Authors:** Shah Saud, Depeng Wang, Shah Fahad, Talha Javed, Mariusz Jaremko, Nader R. Abdelsalam, Rehab Y. Ghareeb

**Affiliations:** ^1^College of Life Sciences, Linyi University, Linyi, China; ^2^Department of Agronomy, Abdul Wali Khan University Mardan, Mardan, Pakistan; ^3^College of Agriculture, Fujian Agriculture and Forestry University, Fuzhou, China; ^4^Division of Biological and Environmental Sciences and Engineering, Smart-Health Initiative and Red Sea Research Center, King Abdullah University of Science and Technology, Thuwal, Saudi Arabia; ^5^Agricultural Botany Department, Faculty of Agriculture (Saba Basha), Alexandria University, Alexandria, Egypt; ^6^Plant Protection and Biomolecular Diagnosis Department, Arid Lands Cultivation Research Institute, The City of Scientific Research and Technological Applications, New Borg El Arab, Egypt

**Keywords:** heavy metals, chromium, oxidative stress, molecular regulation, physiology and biochemistry

## Abstract

In recent years, heavy metals-induced soil pollution has increased due to the widespread usage of chromium (Cr) in chemical industries. The release of Cr into the environment has reached its peak causing hazardous environmental pollution. Heavy metal-induced soil pollution is one of the most important abiotic stress affecting the dynamic stages of plant growth and development. In severe cases, it can kill the plants and their derivatives and thereby pose a potential threat to human food safety. The chromium ion effect on plants varies and depends upon its severity range. It mainly impacts the numerous regular activities of the plant's life cycle, by hindering the germination of plant seeds, inhibiting the growth of hypocotyl and epicotyl parts of the plants, as well as damaging the chloroplast cell structures. In this review article, we tried to summarize the possible effects of chromium-induced stress on plant growth, developmental physiology, biochemistry, and molecular regulation and provided the important theoretical basis for selecting remedial plants in chromium-induced contaminated soils, breeding of low toxicity tolerant varieties, and analyzing the mechanism of plant resistance mechanisms in response to heavy metal stress.

## Introduction

The accumulation of heavy metals in the environment due to modern industrial development is among the most serious pollutions in China and around the world. At present, heavy metal pollution mainly includes mercury (Hg), cadmium (Cd), lead (Pb), chromium (Cr), and arsenic (As). Numerous studies have reported that high concentrations of Cr^6+^ compounds contaminate the soil and hinder the germination and growth of many crops as well as economic plant seeds (Cervantes et al., 2001; Wang and Sheng, 2005; Hayat et al., 2012). Chromium (Cr) is the seventh element on earth that has various valence states of oxidation (Iyaka, 2009), including trivalent (iii) and hexavalent nature (iv). Trivalent chromium is present in its most stable and common valence level. Trivalent chromium is an essential trace element for the human body and plays an important role in the regulation of blood sugar (Zhang Y. et al., 2019), and excessive chromium has an adverse effect on plant photosynthesis and nutrient absorption (Cai et al., 2019). In addition, chromium accumulated in plants enters the body through the food chain, causing dermatitis, bronchitis, tuberculosis, and so on. It also increases the risk of cancer in humans. The toxicity of hexavalent chromium is 100 times more than that of trivalent chromium with serious toxicity (Qianqian et al., 2022), and its high chemical activity, which occurs with the rapid development and use of chemicals in a wide range of applications, is more easily absorbed by plants, resulting in its accelerated release into the environment.

Chromium compounds are heavily influenced by mining, paint manufacturing, petroleum refining, leather tanning, wood preservation, textile manufacturing, pulp processing, and biocide development, and large-scale industrial operations involving chromium lead to extreme environmental pollution (Haider et al., 2022). It is estimated that more than 2,000 tons of chromium enter natural water sources through liquid waste from leather factories every year. The chromium concentration in these waste liquids could be up to 2,000 ~ 5,000 mg L^−1^, which far exceeds the allowable maximum chromium threshold concentration of 2 mg L^−1^ (Adrees et al., 2015; Wei et al., 2022). In the United States, soil chromium concentrations in chromium alloy production areas have reached 25.9 g·kg^−1^ (Zayed and Terry, 2003; Ao et al., 2022). The situation of chromium pollution is not optimistic and ~15% of products in the national economy are related to chromium salts. Each ton of chrome salt products emits more than 2.5 tons of chrome slag, and Na_2_CrO_4_ and CaCrO_4_ in chrome slag are the main components that pollute the environment (Chapman, 2012; Liu et al., 2016; Zhang et al., 2017). Currently, more than 20 million hm^2^ of cultivated land in China is contaminated by heavy metals such as chromium (Batley, 2012; Zhucheng and Lin, 2020).

**Figure 1:**
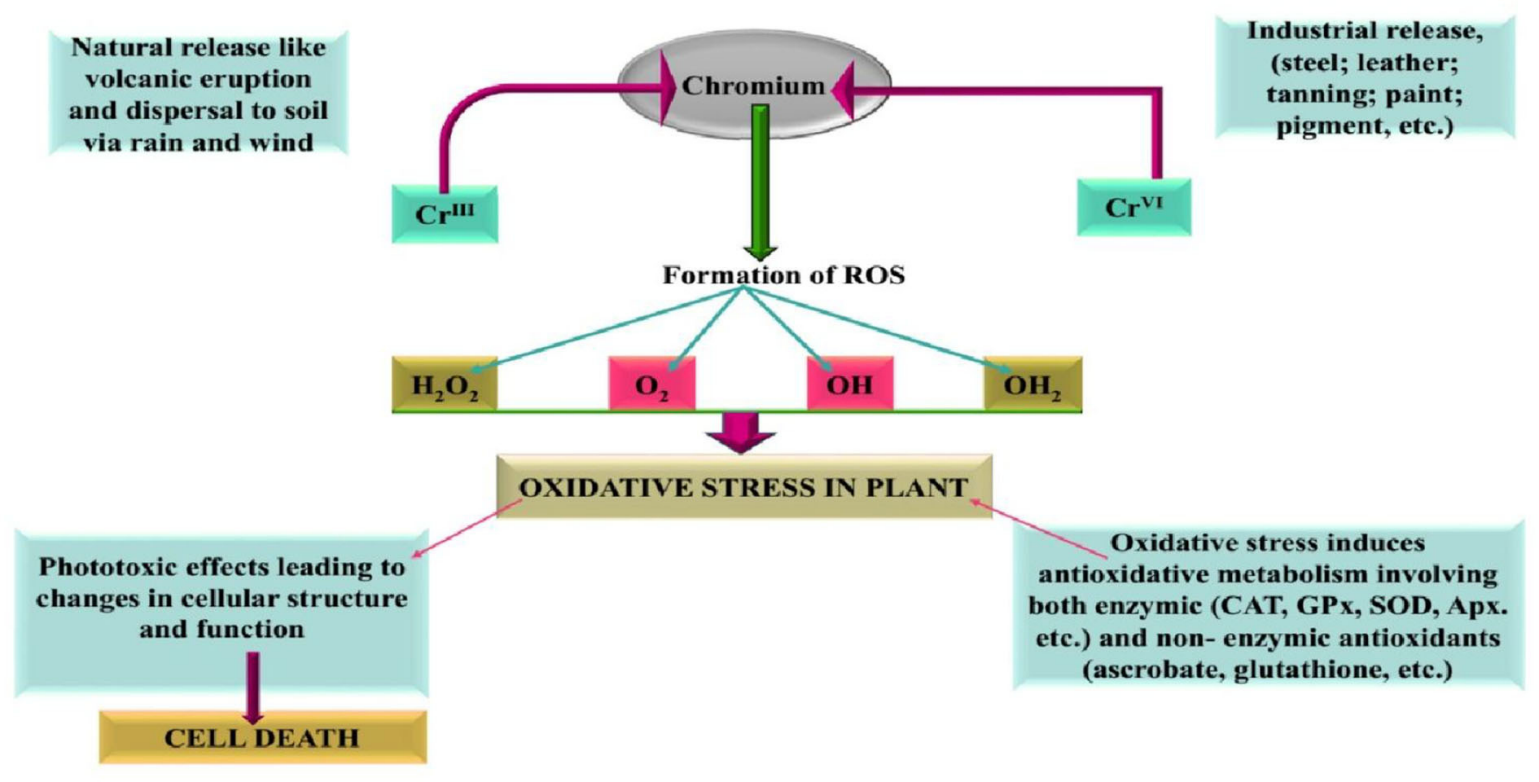
Different sources of chromium and their involvement in including oxidative stress in plants (Panda and Choudhury, 2005).

According to the National Survey Bulletin of the State of Soil Pollution, jointly released by the Department of Environmental Protection and the Department of Land and Resources in 2014, soil in the 14 provinces of the southern China region show higher levels of heavy metal pollution. Pollution from mining, heavy metal processing, and production is particularly severe (Cheng, 2003; Bautista et al., 2013), and it was found that Cd, As, Cr, and other harmful metals account for more than 50 % of the pollution of arable land in an area of 50 km^2^ in Chengdu district, China. The Cr concentration in Chengdu garbage is 35.39–179.63 mg·kg^−1^, which exceeds the soil environmental quality standard. It was found that the detection rate of chromium is 100 % and the excess rate of chromium content is 0.9% after analyzing the soil samples from the plains of Chengdu (Cheng, 2003; Ding et al., 2016; Seneviratne et al., 2017; Li et al., 2018). These data suggest that the state of chromium pollution in other provinces and cities should not be underestimated. Soil contaminated with heavy metals such as chromium further degrade the quality of agricultural crops growing on them and pose a potential threat to human food security. Previous research has suggested that excessive chromium levels severely affected crops of sweet potatoes (*Ipomoeabatatas*) (Gao et al., 2021) and wheat (*Triticum aestivum*) (Wang et al., 2017), and studies on heavy metals have revealed that the threat of chromium contaminated soil is imminent (Figure 1).

The remediation of soil contaminated with heavy metals involves two main aspects of consolidation and activation. The earlier remediation methods were mainly based on excavation and landfill, but their sustainability was not viable, especially with the advancement of science and technology. Further, the treatment of heavy metal soil pollution has now evolved into the use of chemical soil stabilization (Hamon et al., 2002), soil leaching (Huang and Keller, 2020), electrodynamic restoration (Zhang M. et al., 2019), and the use of plant ecological restoration (Lian et al., 2018). The earlier methods were relatively expensive and labor intensive; however, the chemical released can cause secondary pollution of the soil environment. Using plants for heavy metal remediation in the soil is relatively inexpensive, and also has good economic and environmental benefits, which is the key direction for future development and exploitation. To select the appropriate equipment for soil heavy metal remediation, we need to understand the effects of heavy metals on plants. The plant's response to external heavy metal stress is a complex process, involving at least heavy metal stress perception, signaling, and initiation of heavy metal stress resistance mechanisms. The generation of Cr heavy metal stress injuries (Hall and Williams, 2003) is more serious and severely affects plant growth and development, product yield, and quality (Arif et al., 2021).

Cr^6+^ is a typical heavy metal pollutant that can cause numerous diseases. The removal of Cr^6+^ from the environment or the reduction of the highly toxic Cr^6+^ to lesser toxic Cr^3+^ is of great importance in the treatment of chromium exposure (Buchanan-Wollaston and Ainsworth, 1997). Once agricultural surfaces are contaminated with Cr^6+^, then it becomes difficult to clean them and the cost of soil remediation using physical and chemical methods is extremely high. In addition, its toxic effects in plant cells can destroy the cell membranes and internal components of plants, alter the activity of related enzymes in the main body, and then alter the gene expression in plants as well as regulate the bio-synthesis of certain proteins.

**Figure 2:**
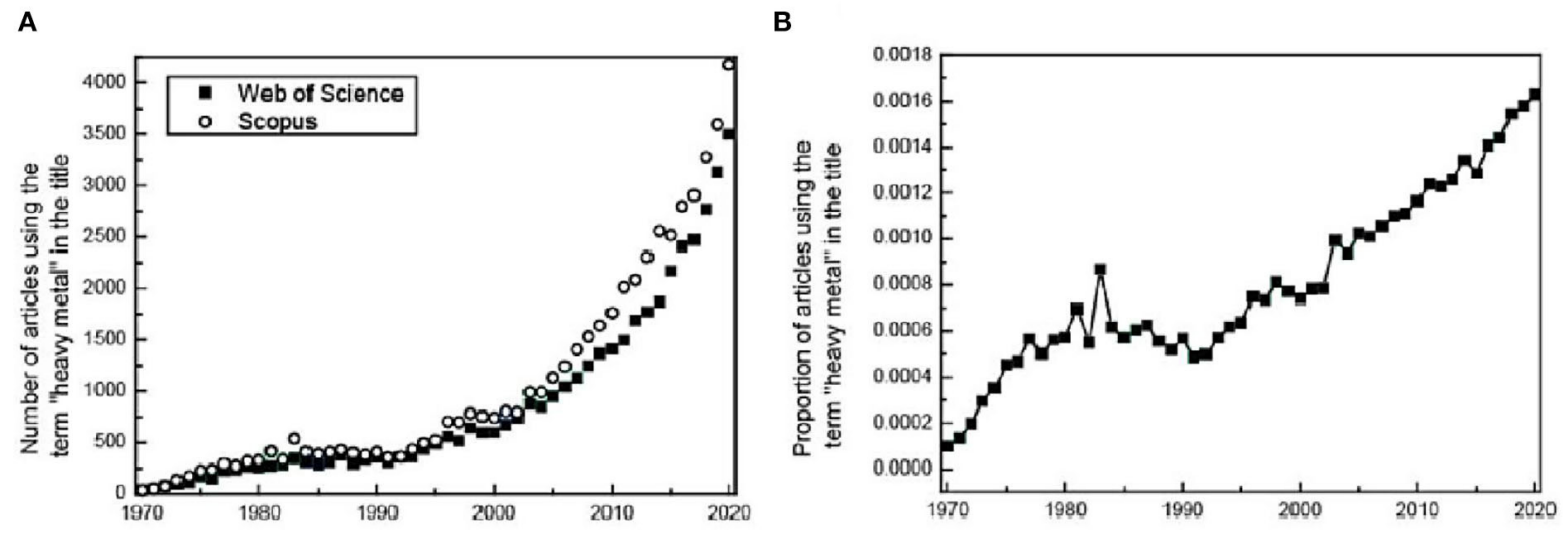
**(A)** Evolution of the number of publications using the term “heavy metal*” in their titles (Scopus and Web of Science, 24 February 2021). Modified and updated from Pourret and Bollinger (2018) and Pourret and Hursthouse (2019). **(B)** Evolution of publications using the term “heavy metals” in their titles (number of articles using the term divided by the total number of all articles published in the year) (Scopus data, searched using “heavy metal*”, accessed 24 February 2021).

In general, plants can prevent chromium from entering the cell through external rejection, such as organic acids, amino acids, and proteins that combine with heavy metals (Chapman, 2007; Osmolovskaya et al., 2018), effectively alleviating the effect of chromium before it enters the cells structures. At the same time, they can enhance the antioxidant defense in plant body defense mechanisms and reduce the damage to ROS caused by chromium stress (Pandey et al., 2005; Ali and Khan, 2018). Research data on the Web of Science and Scopus indicate that the presence of several common heavy metal elements has increased from 0.074% in 2000 to 0.163% in 2020 around the world (Figure 2). However, much of the research is still industry-focused such as materials science, engineering, and physical chemistry (Pourret, 2018; Pourret and Bollinger, 2018; Pourret and Hursthouse, 2019).

The present article effectively summarizes the response mechanisms of plants toward chromium ion stress in multiple aspects, by reviewing the relevant domestic and foreign studies on the effects of chromium stress on plant growth and development, physiology and biochemistry, and molecular regulation. It aims to provide the scientific references and theoretical basis for the development of soil remediation to address heavy metal chromium stress on plants, breeding of low-toxicity plants resistant to heavy metal chromium stress, and discussing the general mechanism of plant heavy metal detoxification. Though the response of plant growth and reaction to chromium stress has been comprehensively understood, there are still some problems to be solved, such as whether there is a difference in chromium tolerance between physiology and biochemistry, and molecular regulation, or the reasons for differences in seed tolerance of different plant varieties. The solution to these problems provides an important theoretical basis for cultivating and screening plant varieties with high resistance to chromium stress.

## Effects of chromium-induced stress on plant growth and development

Plant growth and development are the most important life processes and are influenced by various environmental factors. Chromium is a non-essential element for plants, and its high toxicity affects the germination of plant seeds and the growth and development of roots and shoots.

### The effect of chromium stress on seed germination

Seed germination is the initial stage which is influenced by the heavy metal chromium as plants rely on seeds for reproduction (Chen et al., 2017), and it is also the primary manifestation reflecting the plant's tolerance level to chromium. Induction of stress through hexavalent chromium in the nutrient solution reduced seed germination. At 300 μM, the germination rate decreased significantly from 95.23% of the control to 75.45 %, which may be due to the decrease in enzyme activity triggered by the accumulation of Cr at the stage of seed germination (Duffus, 2002; Ganesh et al., 2006). The higher ROS generated by the Cr treatment may promote the breakdown of retained nutrients in seeds, leading to changes in cell membrane properties (Shafiq et al., 2008; Appenroth, 2010).

It is widely believed that chromium has an inhibitory effect on seed germination. The germination rate was affected by Cr^6+^ concentration in plant species as shown in Table 1. For instance, 100 mg·L^−1^ chromium treated in wheat reduced the germination rate by almost 10%. The same inhibitory effect was observed in *Cucumis melo* (Akinci and Akinci, 2010). Some studies have shown that low levels of chromium promoted seed germination of green vegetables such as *Brassica chinensis, Corchorus spp*, and *Hibiscus sp* (Nikinmaa and Schlenk, 2010; Islam et al., 2014), and then lead to significant inhibition of main root and seed growth with increasing concentrations of chromium in the treatment (Li et al., 2018). In addition, different plant seeds have different tolerance levels to chromium stress concentrations, and different chromium ion concentrations have different effects on plant seed germination. A concentration of 100 M Cr^6+^ and below on rice seed (*Oryza sativa*) has no apparent effect on germination potential, and when the chromium treatment concentration is >25 mg L^−1^, the seeds only germinated without root (Nath et al., 2009). The reason is that chromium sometimes triggers the protease activity resulting in the inhibition of seed germination because the hypocotyl transport is suppressed by the activity of amylase and sugar (Zeid, 2001; Dotaniya et al., 2014b), and in low concentrations, it might promote the α-amylase activity, favoring germination by sugars and thus promoting seed germination (Nayari et al., 1997; Basit et al., 2022a). The low concentration range is mostly between 0 and 10 mg/L.

**Table 1:** Overview of the effect of chromium on plant growth and development.

**Species**	**Cr concentration**	**Effect**	**References**
*Triticum aestivum*	20, 65, 110, 175, and 220 ppm	Inhibition of seed germination	Baruah et al., 2019
*Cucumis melo*	2.5, 5, 10, 25, 50, 75, 100, 200, 300 mg·L^−1^		
*Corchorus spp. and Hibiscus spp*	50, 100, 300, 500 mg·L^−1^		Islam et al., 2014
*Oryza sativa*	0, 0.05, 0.5, 1.0, 10, 25, 50, 100 mg· kg^−1^	Low concentrations promoted seed germination, whereas high concentrations inhibited seed germination	Xie et al., 2018
*Medicago sativa*	0, 100, 200, 300, 400 mg·kg^−1^	Root growth was inhibited	Peralta et al., 2001
*Lablab purpureus*	100, 200, 300 mg·L^−1^		Joutey et al., 2013
*Sesamum indicum*	100, 200, 300 mg·L^−1^		Joutey et al., 2013
*Arabidopsis thaliana*	0, 50, 100, 200 μmol·L^−1^		Wakeel et al., 2018
*Nicotiana langsdorffii*	50 mg·kg^−1^		Del Bubba et al., 2013
*Convolvulus arvensis*	0, 20, 40, 80 mg·L^−1^	Root length decreased; stem elongation was inhibited	Gardea-Torresdey et al., 2004
*Raphanus sativus*	2, 5, 10 mg·L^−1^	Seedling height and root length were reduced	Nath et al., 2009
*Festuca arundinacea*	0, 100, 200, 300, 400 mg·kg^−1^	Plant height, root length, and dry weight were reduced	Daud et al., 2014
*Trifolium repens*	0, 100, 200, 300, 400 mg·kg^−1^		
*Glycine max*	0, 0.05, 0.1, 0.5, 1, 5 mg·L^−1^	Aboveground dry matter was reduced	Turner and Rust, 1971a
*Salvinia minima*	2, 5, 10 mg L^−1^		Prado et al., 2010

Different plant varieties have different maximum thresholds of chromium tolerance (Hayat et al., 2012; Haider et al., 2022). Individual plants have a high tolerance to chromium stress and are chromium tolerant plants, such as B. 20 mg/L. The germination of water bamboo leafy greens seeds (Pandey et al., 2005) was promoted under 100 mg/L chromium stress, and the germination rate under 100 mg/L chromium stress was only 2.20% lower than that of the control. A 25 mg/L chromium stress had a promoting effect on the germination of wheat varieties. The germination rate under 100 mg/L chromium stress was only 8.00 and 0.66% lower than the control (Singh et al., 2021) and Casuarina (Lou et al., 2017; Zulfiqar et al., 2020) which may be related to the difference in plant species and the stress level of Cr^6+^ in the test.

### Effect of chromium stress on underground parts of plants

The root system is the site where plants first encounter the soil's heavy metal ions and it is also the key site to absorb heavy metals which suggest that research into plant root growth is extremely important. In some previous studies, chromium stress had a low-promoting and high-inhibitory effect on plant root growth (Table 2). Cr^6+^-mediated inhibition of root growth may be due to the inhibition of cell division and reduction of cell size in the elongated region (Peralta et al., 2001). It was found that 5 mg·L^−1^ chromium treatment significantly promoted root growth of *Medicago sativa* seedlings. However, when the dose was increased to 20 mg·L^−1^, the promoting effect reversed to inhibit the length of *Convolvulus arvensis*, which decreased by 77% at the same chromium concentration of 20 mg·L^−1^. On the other hand, there was no significant difference in *Convolvulus arvensis* root length after increasing the chromium concentration. Panda and Patra (2000) and Gardea-Torresdey et al. (2004), found that 1 μmol·L^−1^ chromium with simultaneous nitrogen addition also increases the root length of seedlings. Del Bubba et al. (2013) found that treatment with 50 mg/kg^−1^ chromium significantly reduced root biomass of wild-type tobacco (*Nicotiana langsdorffii*), and root accumulation of chromium reached 2,600 μg·g^−1^ in radish (*Brassica campestris*) treated with 1 mg L^−1^ chromium for 21 days and root length was reduced by 80% (Nath et al., 2005; Dotaniya et al., 2014a). Chromium stress also led to a reduction in the number of lateral roots of alfalfa, tall fescue (*Festuca arundinacea*), and white clover (*Trifolium repens*), as well as resulted in yellow and necrotic root tips and root cuttings (Qing et al., 2015; Wakeel et al., 2018). For the same treatment concentration, the inhibitory effect of hexavalent chromium on the root length of onions (*Allium cepa*) was significantly higher than that of trivalent chromium (Liu et al., 1992).

**Table 2:** Overview of the physiological and biochemical effects of chromium on plants.

**Species**	**Cr concentration**	**Effect**	**References**
*Festuca arundinacea Trifolium repens*	0, 100, 200, 300, 400 mg·kg^−1^	Decreased antioxidant enzyme activity	Daud et al., 2014
*Salvinia minima*	2, 5, 10 mg·L^−1^	The contents of photosynthetic pigments (chlorophyll and carotenoids) were not affected	Prado et al., 2010
*Lemna minor*	10, 100, 200 μmol·L^−1^	Leaves turned yellow, and chlorophyll content decreased	Sallah-Ud-Din et al., 2017
*Camellia sinensis*	50, 100, 150 mg·L^−1^	Increase in relative conductivity and malondialdehyde	Pan et al., 2011
*Brassica napus*	400 μmol·L^−1^	Subcellular structural damaged	Gill et al., 2017
*Phaseolus vulgaris*	9.6 × 10^−5^ mol·L^−1^	Chloroplast basal particles increase, the number of sacs decreases, vacuoles size reduced	Vázquez et al., 1987
*Brassica juncea*	0, 0.2, 2, 20 mol·L^−1^	With increasing concentration, SOD, CAT, and APX first increased and then decreased, while GST and GR continuously increased	Pandey et al., 2005
*Kandelia candel*	0, 10, 25, 50, 100, 150, 200, 300 mg·L^−1^	SOD increased first then decreased	Adams et al., 2017
*Zea mays*	0, 0.05, 0.1, 0.25, 0.5, 1.0 mmol·L^−1^	CAT first decreased, then increased, then decreased again	Sharma et al., 2003

In addition, different plants show different degrees of inhibition at the same treatment concentration. For instance, the root length of *Brassica oleracea* was reduced by 31% after 7 days of 100 mg. L^−1^ chromium treatment, while the root length of *Sesamum indicum* was reduced by more than 86% (Peralta et al., 2001). Wakeel et al. (2018) found that the inhibitory effect of hexavalent chromium on the growth of *Arabidopsis thaliana* was enhanced by the stimulation auxin resistance caused by *AUX1* gene expression increasing auxin accumulation in the root tip and affecting its polar transport. The plant root cell wall is the first barrier for plants, preventing heavy metals from entering the body. It consists of three parts: the intercellular layer, the primary wall, and the secondary wall. It is rich in cellulose, pectin, and lignin, and these substances are also rich in hydroxyl and carboxyl groups. When metal ions enter the cell, they combine with these groups and precipitate, reducing the amount of metal ions entering the protoplasmic layer (Eva et al., 2014), in the root cells of white clover, alfalfa, tall fescue, and rice (Zeng et al., 2011). The main storage locations of chromium are cell walls and cytoplasm where the content of plastids such as mitochondria and chloroplasts are low. Wakeel et al. (2020) also found that the chromium content in the vacuoles of aerial plant parts and root cells of chromium-treated vegetables reached 20 ~ 40%, indicating that plants can also transport it in vacuoles to remove it from active ingredient isolate metabolic sites and avoid toxicity to other organelles.

### Effect of chromium stress on above-ground parts of plants

Chromium enters the plants from the root system and is partly transported along with nutrients to the above-ground parts of the plants, which influences the growth of organs such as stems and leaves. The reduction in above-ground biomass after stress is the most intuitive manifestation. Turner and Rust (1971b) and Basit et al. (2022b) found that in hydroponics treated with 0.5 mg L^−1^· chromium and soil cultures treated with 10 mg·L^−1^ chromium could significantly reduce the dry matter content in the aerial parts of soybeans (Glycine max). Turner and Rust (1971b) found similar results in subsequent studies with rice (*Salvinia minima*) and other plants (Prado et al., 2010). Plant height is also significantly affected due to chromium stress. Nath et al. (2005) found that 5 mg L^−1^ of hexavalent chromium can reduce radish seedlings by almost 50%, and stem elongation of *Convolvulus ternata* was also greatly inhibited after hexavalent chromium treatment (Gardea-Torresdey et al., 2004; Rhaman et al., 2020); a similar effect was also observed in alfalfa treated with trivalent chromium (Barton et al., 2000). However, no positive correlation was found between plant dry matter content and plant height. Although 80 mg L^−1^ chromium treatments can greatly inhibit convolvulus stem, elongation did not affect leaf biomass accumulation (Gardea-Torresdey et al., 2004).

It is generally believed that the presence of chromium affects plant photosynthesis and disrupts plant growth. In duckweed (*Lemna minor*), with increasing treatment concentrations, the color of duckweed leaves gradually turned yellow, and the content of chlorophyll and carotenoids decreased significantly (Sallah-Ud-Din et al., 2017). Studies on *Arabidopsis thaliana* showed that chloroplast ribosomal recycling factors played an important role in chloroplast biosynthesis, and changes in maize protein under chromium stress suggested that chloroplast ribosomal recycling factors may be involved in the structure of chloroplast under chromium stress through post-translational modifications (Wang et al., 2013; Grabsztunowicz et al., 2019). Most of the identified chromium stress proteins (72%, 42/58) were predicted to be localized in the chloroplast, and these chloroplast-localized chromium stress proteins were associated with chloroplast structure and function, including the participation in photosynthesis electron transport, chloroplast organization, chlorophyll biosynthesis, and chloroplast redox balance. Although the dry matter content of *Sophora japonicus* continued to decrease with increasing concentration, chromium-treated chlorophyll and carotenoid levels were not affected by chromium. It has been speculated that chromium may also hinder plant growth by interfering with cellular processes other than photosynthesis (Prado et al., 2010). After being treated with 100, 200, and 400 mg/kg^−1^ chromium, the root dry weight of rice was reduced in tillering, booting, and filling phases but the root-to-shoot ratio increased, indicating that the consumed more heavy metal chromium had an obvious inhibition on plant shoots (Xie et al., 2018).

### Toxic effects of chromium on plant roots

Different parts of the plant have different sensitivities to chromium, and the root is the most susceptible part of the plant to chromium poisoning. The changes in root length and root count are important indexes to measure the effect of chromium on the plant (Saxena et al., 2021; Monga et al., 2022). High concentrations of chromium lead to the withering of root cells and separation of the plasma wall, which induces root apex cells to produce a higher frequency of chromosome distortion, resulting in the inhibition of root cell division and differentiation, and reducing the volume and number of root cells. As the root length shortens, the primary root thickens, does not form lateral roots and root hairs, and even does not form a root. Cr^6+^ was found to inhibit the growth of young plant roots more than the growth of sprouts and stems, mainly for the following two reasons: First, chromium's first contact is with the plant's roots, and once plant roots are combined with Cr^6+^ through physical adsorption and chemisorption, chromium could induce roots to produce ethylene stress and transport it to the shoot (Shinwari et al., 2015; Shahid et al., 2017). Stress ethylene causes strong cellular damage, and this damage occurs first in the roots, causing the plant roots to come under chromium stress earlier than the stem and shoot. Secondly, due to the influence of chromium, the structure of the root cell wall of the plant changes along with the content of cellulose. Consequently, the one-OH group and the free carboxyl COO group in the polysaccharide composition increase and bind more Cr^6+^ to confine it at the site, reducing the amount of translocation to the morphological top. As a result, the root system accumulates more chromium than the stem and the shoot (Ao et al., 2022).

### Overall effect of chromium on plant seedling growth

Chromium stress has a tingling effect at low concentrations and inhibits the growth of most plant seedlings at high concentrations. Low chromium concentrations can increase the net rate of photosynthesis and promote plant growth by promoting the electron transport activity of PSII, which can increase the proportion of medulla and outer skin tissue in the root and promote root and root hair growth. A high concentration of chromium hinders water transport, reduces transpiration, affects the uptake of minerals by the root system, and disrupts enzymatic reactions in the plant body, resulting in short stature of the plant, yellowing, and dropping of the leaves, as well as a significant reduction in leaf area and biomass. It was reported that high chromium concentrations could cause permanent plasma wall detachment and water loss in plant tissues (Madrid, 2010; Singh et al., 2021; Srivastava et al., 2021), and irreversible damage to mitochondria, resulting in decreased respiration and even plant cell death. At the same time, it was found that high chromium concentrations caused abnormal stomatal conductivity, reduced intercellular space, and reduced growth and yield of plants (Rath and Das, 2021).

## Impacts of chromium stress on plant physiology and biochemistry

The accumulation of chromium in plants inevitably disrupts their homeostasis, damages cell structure, and affects the dynamic balance of the antioxidant enzyme system.

### Effects of chromium stress on cell membrane permeability in plants

The selective permeability of the cell membrane can prevent the inflow and outflow of extracellular substances, make the intracellular environment relatively stable, and ensure that the biochemical reactions required for life can proceed in an orderly manner. When plants are poisoned by heavy metals, these harmful metal ions damage cell membranes and their selective permeability, thereby greatly increasing permeability, causing membrane lipid peroxidation, and affecting the normal growth and development of plants. Relative conductivity and malondialdehyde are the two most commonly used indicators to measure cell membrane damage among which, malondialdehyde, as the final product of membrane lipid peroxidation, can indirectly assess the damage of plant cell membranes. Draper and Hadley (1990) found that the relative conductivity of *Cyperus malaccensis* leaves increased with increasing convulsive concentration, and the relative conductance of malondialdehyde increased from 500 mg L^−1^ by 255.66 and 185.74% with increasing chromium concentration. The cell membrane structure was significantly destroyed. Pan and Yu (2011) found that the combination of Cr, As, Pb, and Cd could increase the relative conductivity of tea tree leaves (*Camellia sinensis*) and found that combinations of heavy metals treated differently in chromium concentrations changed relative conductivity. According to the area analysis of the orthogonal experiment, chromium has the strongest effect on membrane permeability.

### Damage of plant subcellular structure under chromium stress

When chromium crosses membranes in cells, it inevitably damages the cell's internal structure. Gill et al. (2017) found that rapeseed (*Brassica napus*) produced large black plastoglobules (PG) and increased the volume and number of starch granules (SG) under chromium stress. The chloroplast ruptured and the thylakoid membrane was also damaged after the dwarf bean (*Phaseolus vulgaris*) was treated with chromium; the chloroplasts in the first leaf cells showed more basal granules and the number of thylakoids decreased compared to the control. A large central nucleus appeared in the third leaf cells, which were smaller vacuoles (Eleftheriou et al., 2015).

### Effects of chromium on the enzymatic anti-oxidant system of plants

Under normal circumstances, the synthesis and decomposition of reactive oxygen species (ROS) in plants would be in dynamic balance; but when exposed to chromium stress, the cellular redox balance is disrupted, and the ROS level in plants increases significantly, which induces hyperoxia affects the production of antioxidant enzymes such as superoxide dismutase (SOD), catalase (CAT), peroxidase (POD), and ascorbate peroxidase (APX), which are used to resist the toxic effects of heavy metals, which in turn affect plant growth. The antioxidant enzyme systems of different plants react differently to chromium stress (Monga et al., 2022). When mustard (*Brassica juncea*) was treated with different concentrations of chromium, it was found that the activities of SOD, CAT, and APX in roots and leaves first increased and later decreased (Pandey et al., 2005; Hübner et al., 2010), while glutathione transferase (GST) and glutathione reductase (GR) showed an increasing trend. The POD, CAT, and SOD that were treated with hexavalent chromium in duckweed (*Lemna minor*) (Lopez-Luna et al., 2009; Chen et al., 2015), *Arabidopsis* plants and *Vallisneria spiralis* (Xu and Deng, 2012; Elisa and Bartoli, 2013) showed similar expression trends. This is based on the fact that under chromium stress, chromium ions act as a signaling molecule to initiate the plant's antioxidant defense mechanism, and as stress damage increases, antioxidant enzyme activity continues to decrease (Mallick et al., 2010). However, the CAT content in maize (*Zea mays*) first showed a trend of decreasing (Table 3), then increasing and then decreasing with chromium treatment concentration which may be due to the iron porphyrin contained in the CAT structure, and the interaction between chromium and iron in the metabolic pool resulting a reduction in its activity. It is also possible that the presence of chromium affects the availability of active iron (Sharma et al., 2003).

**Table 3:** Effects of chromium metal on different physiological processes in plants.

**Plant Species**	**Physiological response**	**References**
*Camellia sinensis*	Increased SOD and CAT activities	Tang et al., 2014
*Capsicum annuum*	Increased carotenoid content	Oliveira, 2012
*Chamomilla recutita*	Increased MDA level	Kováčik et al., 2013
*Echinochloa colona*	Increased CAT and POD activities	Samantaray et al., 2001
*Kandelia candel*	Increased MDA content, and activities of CAT and SOD	Rahman et al., 2010
*Ocimum tenuiflorum*	Increased proline level	Rai et al., 2004
*Oryza sativa*	Increased POD activity	Ma et al., 2016
*Oryza sativa*	Increased ethylene synthesis	Trinh et al., 2014
*Oryza sativa*	Increased CAT and SOD activities	Zhang et al., 2010
*Oryza sativa*	Increased POD activity	Xu et al., 2011
*Phaseolus vulgaris*	Decreased carotenoids	Aldoobie and Beltagi, 2013
*Pisum sativum*	Decreased APX activity	Duhan, 2012
*Pterogyne nitens*	Increased spermidine level	Paiva et al., 2014
*Raphanus sativus*	Increased glycine-betaine content	Choudhary et al., 2012
*Triticum aestivum*	Increased MDA contents	Ali et al., 2015
*Triticum aestivum*	Increased lipid peroxidation	Zhang et al., 2010
*Vigna radiata*	Decreased glutathione level	Shanker et al., 2004
*Zea mays L*.	Increased SOD and GPX activities	Maiti et al., 2012
*Zea mays L*.	Increased lipid peroxidation and H_2_O_2_ content	Maiti et al., 2012

## Impacts of chromium-induced stress on molecular regulation of plant mechanism

With the rapid development of natural sciences, people began to discover that the mechanism of plant resistance is closely related to their own gene expression. The study of the physiological direction cannot solve all scientific problems. Currently, scientists have explored the effects of heavy metal chromium on plants from a molecular biology perspective and found that gene expression, regulation, and protein synthesis in the body change accordingly when plants respond to chromium stress (Table 4). With the development of molecular biology, some genes related to chromium resistance have been reported. In sugarcane (*Saccharum spp*. hybrid), Jain et al. (2016) found that the expression of the i-gene of the metallothionein (*MT*) gene was found to increase significantly in stem and leaf under chromium stress. It is consistent with previous reports that MT protein can bind metals and detoxify excess metal ions (Gepstein et al., 2003; Kim et al., 2007). Gill et al. (2017) found that the genes *BnaA08g16610D, BnaCnng19320D*, and *BnaA08g00390D* were overexpressed in rapeseed after chromium treatment.

**Table 4:** Related genes or proteins induced by chromium treatment in plants.

**Species**	**Cr concentration**	**Related genes/proteins**	**Functions of related genes/proteins**	**References**
*Saccharum* spp. hybrid	0, 10, 15 mg·L^−1^	Metallothionein	Metal binding, detoxification of excessmetal ions	Jain et al., 2016
*Brassica napus*	400 μmol·L^−1^	*BnaA08g16610D*, *BnaCnng19320D*, *BnaA08g00390D*, *BnaA04g26560D*, *BnaA02g28130D*, and *BnaA02g01980D*	Encoding nucleic acids; transition metal ion binding protein; water molecule transmembrane transport, etc	Sallah-Ud-Din et al., 2017
*Miscanthus sinensis*	0, 50, 100, 200, 300, 500, 750, 1,000 μmol·L^−1^	Carbon and nitrogen metabolism-related proteins, heavy metal inducing proteins, and enzyme proteins	Carbon and nitrogen metabolism, molecular chaperone, and energy metabolism	Sharmin et al., 2012
•*Hordeum vulgare* •*Brassica napus*	0, 10, 30, 50, 100 μg·mL^−1^	Chitinase	It may be related to plant cross tolerance	Jacobsen and Hauschild, 1992
*Nicotiana tabacum*	0, 100 μmol·L^−1^	Dehydrin, superoxide dismutase, mitochondrial malate dehydrogenase, etc	Stress response, energy metabolism, RNA binding, metabolism, etc	Bukhari et al., 2015
*Oryza sativa*	2, 200 μmol·L^−1^	NADP Isocitrate dehydrogenase, S-adenosyL-methionine synthetase, etc	Photosynthesis, signaling molecules, and molecular chaperones	Zeng et al., 2014
*Callitriche cophocarpa*	1 mmol·L^−1^	NAD(P)H-dependent dehydrogenase *FQR1*	*In vitro* quinone reductase activity and catalytic transfer of elect	Kaszycki et al., 2018
*Brassica chinensis*	0, 10, 50, 100 μmol·L^−1^	Phytochelatin	Chelated heavy metals	Wakeel et al., 2020

These genes encode nucleic acids, transition metal ion binding proteins kinase activity, phosphotransferase activity, and molecular transporter genes *BnaA04g26560D, BnaA02g28130D*, and *BnaA02g01980D* were identified as responsible for transmembrane transport of water molecules in the presence of chromium. The use of transgenic technology to introduce non-plant resistance genes could decrease chromium uptake by plants. Jin et al. (2001) transferred the reductase gene of a heavy metal-reducing bacterium- Pseudomonas aeruginosa into tobacco (*Nicotiana tabacum*), which could effectively reduce the content of hexavalent chromium in tobacco plants. In addition, when the rat glucocorticoid receptor gene (GR receptor) was transferred to tobacco, the amount of chromium absorbed by the plants was significantly reduced (Fuoco et al., 2013; Lopez-Luna et al., 2016). With the development of high-throughput sequencing technology, it is still necessary to further discuss the mining of important chromium stress response genes and the construction of expression networks among a large number of sequencing results.

The miRNA is a class of exogenous non-coding small RNA that regulates gene expression by inducing cleavage of target mRNA or inhibition of translational at the transcriptional and post-transcriptional levels (Turner et al., 2012). Such small RNA also plays an important role in plant response to heavy metal stress (Zhou et al., 2012). Forty-one conserved miRNA families were identified in chromium-treated tobacco (Bukhari et al., 2015), of which 57 miRNAs from 26 families were up-regulated, 8 miRNAs from the miR166 family were downregulated, and 29 new miRNA families were identified where 14 were differentially expressed under chromium stress. The COG functional class analysis of these miRNAs showed that some predicted miRNA target transcripts were responsive to biological and abiotic stresses, including miR166 target genes derived from the III HD-ZIP family which regulates lateral rooting in Arabidopsis (Bowman, 2004). These results suggest that it may play a similar role in tobacco chromium tolerance.

In addition, chromium stress can also alter the expression of certain proteins in plants. In recent proteomics studies (Sharmin et al., 2012), 36 proteins were found differentially expressed in the roots of *Miscanthus sinensis* under chromium stress, of which 13 were up-regulated, 21 were downregulated, and 2 new proteins were induced. These proteins turned out to be well-known carbon and nitrogen metabolism-related proteins, molecular chaperone-like heavy metal-inducing proteins, and some enzymes such as inositol mono-phosphatase, nitrate-reducing enzymes (reductase), adenine phosphoribosyl transferase, and other enzyme proteins. In barley (*Hordeum vulgare*) and grapes (B*rassica napus*), hexavalent chromium increased chitinase activity (Hewitt, 1953; Jacobsen and Hauschild, 1992), which may be related to the cross-tolerance of plants. In transgenic tobacco overexpressing fungal chitinase, the plant not only increased its resistance to fungal infections but also to salinity and metal ion stress (Hodson, 2004; De Las Mercedes Dana et al., 2006), although the relationship between chitinase and plant metal tolerance is still unclear.

However, the role of metal-specific chitinase may help explain the mechanism of chromium detoxification in plants. Bukhari et al. (2015) found that under chromium stress, dehydrin (DHN), mitochondrial processing peptidase-like (MPP), adenine phosphoribosyl transferase, superoxide dismutase, and mitochondrial malate dehydrogenase (mitochondrial malate dehydrogenase (MDH) in plants were expressed differently. These proteins were further classifications of into four categories based on their function such as related to stress response, energy metabolism, RNA binding, and metabolism. In rice, NADP isocitrate dehydrogenase, heat shock protein 90 (Hsp90), glyoxalase I (glyoxalase I), protein-glycosylated peptides (reversibly the expression of the glycosylated polypeptide (RGP), S-adenosyl-methionine synthetase (SAMS), glutamine synthetase (glutamine synthetase), and other proteins were up-regulated (Zeng et al., 2014). Kaszycki et al. (2018) found that the submerged plant Callitriche cophocarpa expressed a novel NAD(P)H-dependent dehydrogenase FQR1, thought to be a detoxifying protein, after treatment with hexavalent chromium to protect cells from oxidative damage in response to oxidative stress. It exhibited quinone reductase activity *in vitro* and catalyzed the transfer of two electrons from NAD(P)H to a variety of substrates, including hexavalent chromium. This enzyme is specifically induced by chromate and cannot be produced under salinity and oxidative stress.

Plant-chelating peptides (phytochelatin, PCs) are a type of heavy metal chelating proteins that are widely distributed in plants. Catalyzing heavy metal-induced glutathione synthesis (Zenk, 1996; Handa et al., 2018), after chelating with heavy metals, they can be further transported to vacuoles to reduce the intracellular concentration of free heavy metal ions (Brunetti et al., 2015) and they play an important role in heavy metal detoxification. It was found that hexavalent chromium could significantly increase the PC content in the aerial parts of green vegetables, and varieties high in PC content can accumulate more chromium in roots and cell walls, and significantly improve their tolerance (Pan et al., 2011). Glutathione (GSH), which is widely distributed in organelles such as cytoplasm, chloroplasts, and mitochondria, is a direct substrate for the synthesis of PCs. The sulfhydryl group in its chemical structure has the unique property of stabilizing mercapto-metal bonds. This stable binding form combined with higher water solubility allows GSH to better eliminate the toxicity of a variety of heavy metals (Zlobin et al., 2017). After heavy metal stress, the content of GSH in plants decreased due to the increased amount of PC synthesis. Pan et al. (2011) showed that increased PC content is much higher than decreased GSH content, indicating that hexavalent chromium can also stimulate plants to synthesize new GSH. Furthermore, exogenous application of GSH reduced the concentration of free chromium ions and aerial chromium ions in rice roots, indicating that GSH can inhibit chromium ion transport (Zeng et al., 2012), alleviate the damage to plants caused by chromium, and reverse the damage to physiological functions and cellular structure (Hou et al., 2014; Gill et al., 2017).

The uptake of heavy metals in plants affects the normal metabolism of plants. To reduce its toxic effects, plants also excrete organic acids with a high affinity for heavy metals to combine with heavy metal ions to form chelates, which can be divided into external and internal chelation (Oliveira, 2012; Liu et al., 2018). External chelation refers to plants that secrete organic acids through their roots into the rhizosphere that combine with metal ions to alter their mobility and solubility in the medium (Zeng et al., 2008). Increasing and prolonging chromium content in the culture medium treatment time increased the secretion of organic acids such as oxalic acid, citric acid, and malic acid, which promoted the absorption of chromium ions by rice. The exogenous application of citric acid to duckweed (*Lemna minor*) also promoted the absorption of chromium ions by rice (Sallah-Ud-Din et al., 2017).

The concentrations of citric, formic, lactic, malic, oxalic, and succinic acid in the rhizosphere of *Silene vulgaris* were significantly increased at 100 mg·kg^−1^ chromium treatment, but the concentrations varied with different genotypes (García-Gonzalo et al., 2017). Internal chelation mainly refers to the combination of organic acids produced by the plant's own metabolism with heavy metal ions absorbed by the plant, transforming them into non-toxic or low-toxic forms and alleviating their toxic effects on plants (Hall, 2002). Citric acid, malic acid, and oxalic acid are the main organic acids used to chelate metals in plants; however, it is still controversial whether organic acids can detoxify heavy metals in plants. Regardless, in the study by Juneja and Prakash (2005), it was found that citric acid and malic acid were the main complexing agents of trivalent chromium in corn xylem sap, and the complication of trivalent chromium was beneficial to the dissolution and migration of chromium and it was considered that these organic acids may indeed have a detoxification effect on plants.

## Summary and future perspectives

In summary, while research on the effects of chromium on plants compared to other heavy metal ions started earlier, it has not been deep enough to study the plants under heavy metal chromium stress response mechanisms and degradation-related genes and proteins or to investigate which is clearly related to the signal transduction network. Further work is required for screening appropriate plants to cultivate on the soil contaminated by heavy metals. There are two approaches to restoring vegetation in heavy metal-contaminated land by using plants for ecological restoration. This involves finding plants with strong qualities of repulsing heavy metal ions or accumulating them underground, thereby effectively utilizing the abandoned and polluted land, but also avoiding the potential risks of using the above-ground part. The use of hyperaccumulators in soil treatments contaminated with heavy metals to restore ecological function is quite common. Hyperaccumulator plants refer to plants whose absorption of heavy metal ions is more than 100 times that of ordinary plants, and whose leaf-to-root ratio of heavy metal content is >1 (Baker et al., 2000). For chromium, the plant absorption threshold should be 300 μg·g^−1^ (Garcíahernández et al., 1998; Van Der Ent et al., 2013). Current studies have identified that plants such as *Leersiahexandra* (Zhang et al., 2007), *Prosopis laevigata* (Buendía-González et al., 2010; Gill et al., 2016), and *Spartina argentinensis* (Redondo-Gómez et al., 2011) can be hyperaccumulators of chromium. However, existing hyperaccumulator plants have low biomass and economic benefits, so they cannot be used in a variety of practical remediation projects.

Therefore, the researchers believe that further selection and breeding of plants can not only fulfill the ecological restoration function but can also have a higher biomass yield and heavy metal ions can accumulate in the above-ground part, which can effectively and economically control the heavy metal contamination of land. Previous reports of Miscanthus, which are widely distributed in different countries, have rich wild resources, developed root systems, and large biomass, and can absorb organic pollutants, heavy metals, and other solid matter to prevent soil erosion, and promote carbon deposition. They can be used for ecological upgrading of heavy metals as an energy plant, and they also have a very high economic value.

**Figure 3:**
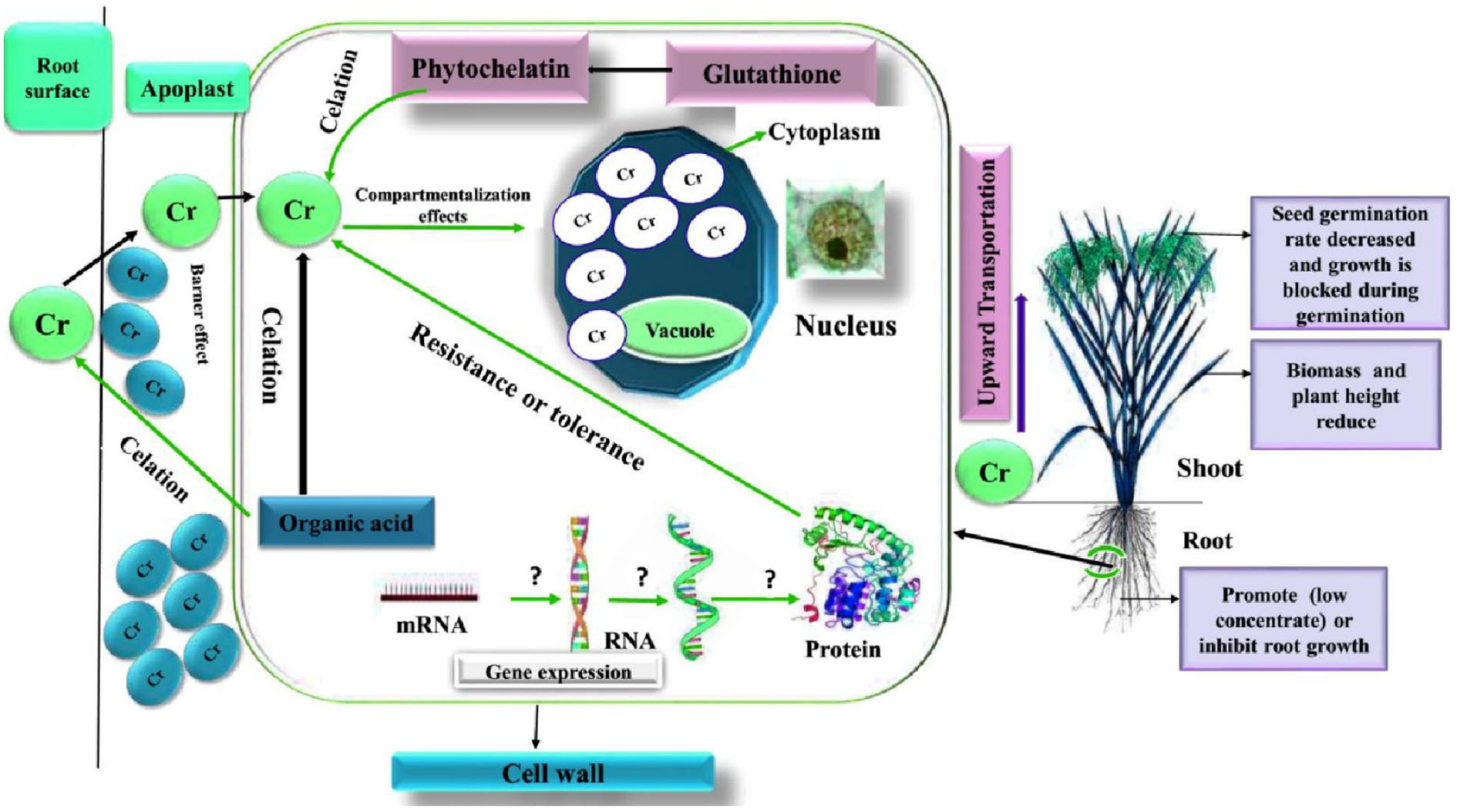
Schematic diagram of the influence of heavy metal chromium on plant growth, physiology, and molecular regulation (Shanker et al., 2005).

As an industrially widespread heavy metal, chromium is one of the main sources of environmental pollution. Its high toxicity is closely related to its rapid permeability through biofilm and subsequent interaction with proteins and nucleic acids in plants. For some plants, the effects of chromium listed in Tables 3, 4 [from the point of view of independent seed germination, external performance, aboveground and root growth, internal regulatory genes, and the change in the protein (Figure 3)], show that the plant's defense against heavy metal chromium, absorption, resistance, and detoxification mechanism are very complicated and that there are major differences between different plants in their response to chromium stress. In addition, previous studies have shown that chromium also has some effect on the absorption of other heavy metals (Turner and Rust, 1971b; Moral et al., 1996). In general, the existing research has gradually deepened from physiology to molecular direction. Genomics, proteomics, and metabolome research have become an important direction in the study of the mechanism of heavy metal chromium tolerance in plants, and many issues urgently need further research.

## Author contributions

SS and DW wrote the initial draft of the manuscript. TJ designed the whole idea of the review article. TJ, MJ, NA, and RG improved the initial draft of the manuscript and also gathered the data. SF undertook the graphical visualization and proofreading of the final version of the manuscript. All authors have read and agreed to the published version of the manuscript.

## Funding

This research was supported by the National Natural Science Foundation of China (Projects No. 32001468).

## Conflict of interest

The authors declare that the research was conducted in the absence of any commercial or financial relationships that could be construed as a potential conflict of interest.

## Publisher's note

All claims expressed in this article are solely those of the authors and do not necessarily represent those of their affiliated organizations, or those of the publisher, the editors and the reviewers. Any product that may be evaluated in this article, or claim that may be made by its manufacturer, is not guaranteed or endorsed by the publisher.
